# Enhanced Palmitate-Induced Interleukin-8 Formation in Human Macrophages by Insulin or Prostaglandin E_2_

**DOI:** 10.3390/biomedicines9050449

**Published:** 2021-04-21

**Authors:** Janin Henkel, Julia Klauder, Meike Statz, Anne-Sophie Wohlenberg, Sonja Kuipers, Madita Vahrenbrink, Gerhard Paul Püschel

**Affiliations:** 1 Department of Nutritional Biochemistry, Institute of Nutritional Science, University of Potsdam, D-14558 Nuthetal, Germany; manowsky@uni-potsdam.de (J.K.); statz@uni-potsdam.de (M.S.); wohlenberg@uni-potsdam.de (A.-S.W.); kuipers@uni-potsdam.de (S.K.); vahrenbrink@uni-potsdam.de (M.V.); gpuesche@uni-potsdam.de (G.P.P.); 2 Department of Nutritional Biochemistry, Faculty of Life Sciences: Food, Nutrition and Health, University of Bayreuth, D-95326 Kulmbach, Germany

**Keywords:** macrophages, insulin, prostaglandin E_2_, interleukin-8, inflammation

## Abstract

Macrophages in pathologically expanded dysfunctional white adipose tissue are exposed to a mix of potential modulators of inflammatory response, including fatty acids released from insulin-resistant adipocytes, increased levels of insulin produced to compensate insulin resistance, and prostaglandin E_2_ (PGE_2_) released from activated macrophages. The current study addressed the question of how palmitate might interact with insulin or PGE_2_ to induce the formation of the chemotactic pro-inflammatory cytokine interleukin-8 (IL-8). Human THP-1 cells were differentiated into macrophages. In these macrophages, palmitate induced IL-8 formation. Insulin enhanced the induction of IL-8 formation by palmitate as well as the palmitate-dependent stimulation of PGE_2_ synthesis. PGE_2_ in turn elicited IL-8 formation on its own and enhanced the induction of IL-8 release by palmitate, most likely by activating the EP4 receptor. Since IL-8 causes insulin resistance and fosters inflammation, the increase in palmitate-induced IL-8 formation that is caused by hyperinsulinemia and locally produced PGE_2_ in chronically inflamed adipose tissue might favor disease progression in a vicious feed-forward cycle.

## 1. Introduction

White adipose tissue is a specialized lipid storage organ in which humans, like other mammals, can stockpile vast amounts of excess energy that they ingest if food intake exceeds caloric demand. Triglycerides are kept as a reserve for periods of poor food supply. If, however, as in industrialized countries, food supply chronically surpasses the energy requirement, white adipose tissue is driven to its functional limits. The excessive hypertrophic and hyperplastic expansion of white adipose tissue in overweight or obese patients is therefore accompanied by chronic, low-level inflammation [1]. Resident and additional infiltrating macrophages, which form crown-like structures around dysfunctional and dying adipocytes, are central players in this inflammation. They can be activated by danger-associated molecular patterns (DAMPs) that are released from failing adipocytes and by pathogen-associated molecular patterns (PAMPs) such as lipopolysaccharides from Gram-negative bacteria, which have been shown to be increased in the plasma of overweight patients consuming high-fat diets due to increased production by an altered gut microbiome and an enhanced uptake [2,3,4,5]. Toll-like receptors (TLRs), in particular TLR-4, are activated by these signals and trigger the release of pro-inflammatory mediators such as cytokines, chemokines, and prostaglandin E_2_ (PGE_2_) from macrophages. In addition to DAMPs and PAMPs, saturated fatty acids, in particular palmitate, have been reported to trigger an inflammatory response in macrophages [6,7], possibly in a TLR4-dependent manner. Palmitate levels have been found to be elevated in the plasma of overweight patients [8,9]. The insulin-dependent reduction in plasma palmitate levels was impaired in particular in patients with abdominal obesity [10]. Furthermore, recent evidence suggests that the pro-inflammatory response may be fostered or even triggered by insulin [11,12], the plasma concentration of which is elevated in overweight or obese patients in an attempt to compensate for the insulin resistance that ensues from the excessive expansion of adipose tissue. Thus, macrophages in pathologically expanded dysfunctional white adipose tissue are exposed to a mix of potential modulators of inflammatory response, including fatty acids, PGE_2_, and insulin. The current study, therefore, addressed the question of how palmitate might interact with insulin or PGE_2_ in the induction of the chemokine interleukin-8 (IL-8, CXCL-8). IL-8 is an important chemotactic activator of local inflammatory responses [13] and a potent inductor of insulin resistance in human adipocytes [14] whose concentration is increased in the circulation of obese patients compared to normal-weight controls [15].

## 2. Materials and Methods

All chemicals were of analytical or higher grade and obtained from local providers unless otherwise stated.

### 2.1. Cultivation of Human Macrophage Cell Line THP-1

The human monocytic cell line THP-1 was cultivated in medium RPMI1640 with 10% heat-inactivated FCS and 1% antibiotics (all from Biochrom AG, Berlin, Germany) and seeded in 35 mm diameter culture plates with 1 × 10^6^ cells per plate. Monocytes were differentiated into macrophages by the addition of 100 ng/mL phorbol-12-myristate-13-acetate (PMA) (Sigma-Aldrich, Taufkirchen, Germany) for 24 h. After removing the medium, macrophages were washed with RPMI1640 and incubated in RPMI1640 without PMA, supplemented with 0.5% serum and 1% antibiotics for 24 h. For cell experiments and the preparation of supernatants, macrophages were stimulated for another 24 h with 100 nM of insulin (Sigma-Aldrich), 100 µM of palmitate (dissolved under alkaline conditions and coupled to bovine serum albumin (BSA), as described previously [16], or a respective control), 10 µM of PGE_2_ (Enzo Life Sciences, Lörrach, Germany), or 1 µM of agonists (17-phenyl trinor prostaglandin E_2_ for EP1/3, 19-(R)-hydroxyprostaglandin E_2_ for EP2 and CAY10598 for EP4, all Cayman Chemical, Ann Arbor, Michigan, USA) or EP4-antagonist (ONO AE3-208, Cayman Chemical, Ann Arbor, Michigan, USA). The used concentrations were consistent through all experiments. Cells and supernatants were shock-frozen in liquid nitrogen and stored at −70 °C for further analysis.

### 2.2. Real-Time RT-PCR Analysis

RNA isolation, reverse transcription, and qPCR were performed as previously described [17]. Oligonucleotide sequences are listed in Supplementary Table S1. Results are expressed as relative gene expression normalized to the expression levels of reference gene β-actin according to the formula: fold induction = 2 ^(control−treated) gene of interest^/2 ^(control−treated) reference gene^.

### 2.3. Determination of IL-8 and PGE_2_

Cell culture supernatants were analyzed with enzyme-linked immunoassay kits for the determination of IL-8 (Life Technologies, Darmstadt, Germany) or PGE_2_ (Cayman Chemical, Ann Arbor, Michigan, USA) according to the manufacturer’s instructions.

### 2.4. Statistical Analysis

The statistical significance of differences was determined by Student’s *t*-test, one-way-ANOVA, or two-way-ANOVA with Tukey’s posthoc test for multiple comparisons, as appropriate, using GraphPad Prism v8 for Windows (GraphPad Software, La Jolla California, USA). Differences with *p* ≤  0.05 were considered statistically significant. For details, see the legends of the figures.

## 3. Results

### 3.1. Insulin-Enhanced Palmitate-Dependent Induction of IL-8 in THP-1 Macrophages

**Figure 1:**
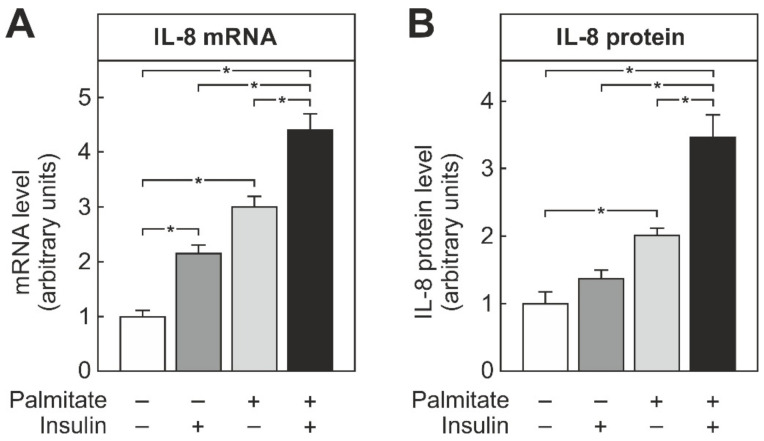
IL-8 induction by palmitate and insulin in THP-1 macrophages. THP-1 cells were differentiated into macrophages by PMA treatment as detailed in the methods section and then incubated with 100 nM insulin, 100 µM palmitate, or both for 24 h, as indicated. mRNA levels (**A**) were determined by RT-qPCR, and IL-8 protein (**B**) was quantified by ELISA. Data were normalized to the average induction under all conditions. Values are means ± SEM of at least 11 independent experiments. Statistics: * *p* < 0.05, two-way ANOVA with Tukey’s post hoc test.

THP-1 cells were differentiated into macrophages as described in the methods section. Subsequently, they were incubated for 24 h in the presence of insulin, palmitate, or a combination of both, as indicated (Figure 1), and the IL-8 expression was determined by RT-qPCR or ELISA. Both insulin and palmitate induced the IL-8 mRNA significantly, by 2.5-fold and 3-fold, respectively. The induction of the IL-8 mRNA was even more pronounced when the cells were exposed to a combination of insulin and palmitate (Figure 1A). Similarly, incubation with palmitate increased the secretion of IL-8 into the cell culture supernatant significantly, by about 2.5-fold. While insulin on its own did not increase IL-8 protein secretion into the cell culture supernatant, it significantly enhanced the palmitate-induced secretion of IL-8 (Figure 1B). 

To exclude that contamination with lipopolysaccharide (LPS) was the reason for the palmitate-dependent induction of IL-8, a set of experiments was repeated with polymyxin B, which binds and inactivates LPS. Polymyxin B did not inhibit the palmitate-dependent IL-8 induction, excluding LPS contamination (not shown).

### 3.2. Palmitate- and Insulin-Dependent Induction of PGE_2_ Synthesis in THP-1 Macrophages

**Figure 2:**
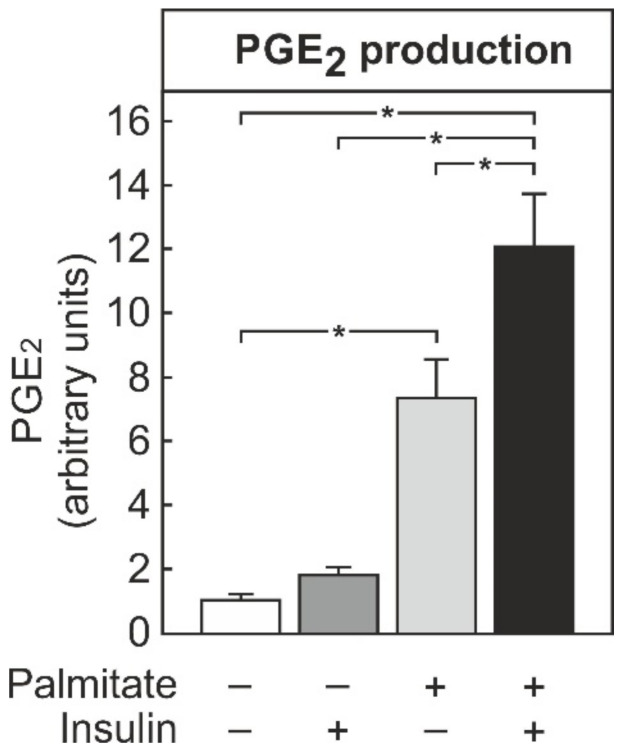
Stimulation of PGE_2_ formation by palmitate and insulin in THP-1 macrophages. THP-1 cells were differentiated into macrophages by PMA treatment, as detailed in the methods section, and then incubated with 100 nM insulin, 100 µM palmitate, or both for 24 h, as indicated. The PGE_2_ concentration in the cell culture supernatants was quantified by ELISA. Data were normalized to the average induction under all conditions. Values are means ± SEM of 6 independent experiments. Statistics: * *p* < 0.05, two-way ANOVA with Tukey’s post hoc test.

A combination of palmitate and insulin significantly induced cyclooxygenase-2 (COX-2) and microsomal PGE synthase-1 (mPGES-1), two inducible key enzymes for the PGE_2_ production in macrophages during an inflammatory response (Supplementary Table S2). Therefore, the PGE_2_ secretion into the cell culture supernatant after the exposure of THP-1 macrophages to insulin and palmitate was determined (Figure 2). Palmitate significantly increased PGE_2_ production in THP-1 macrophages. Although insulin did not elicit PGE_2_ production on its own, it significantly enhanced palmitate-induced PGE_2_ production.

### 3.3. PGE_2_-Dependent Modulation of IL-8 Formation in THP-1 Macrophages

**Figure 3:**
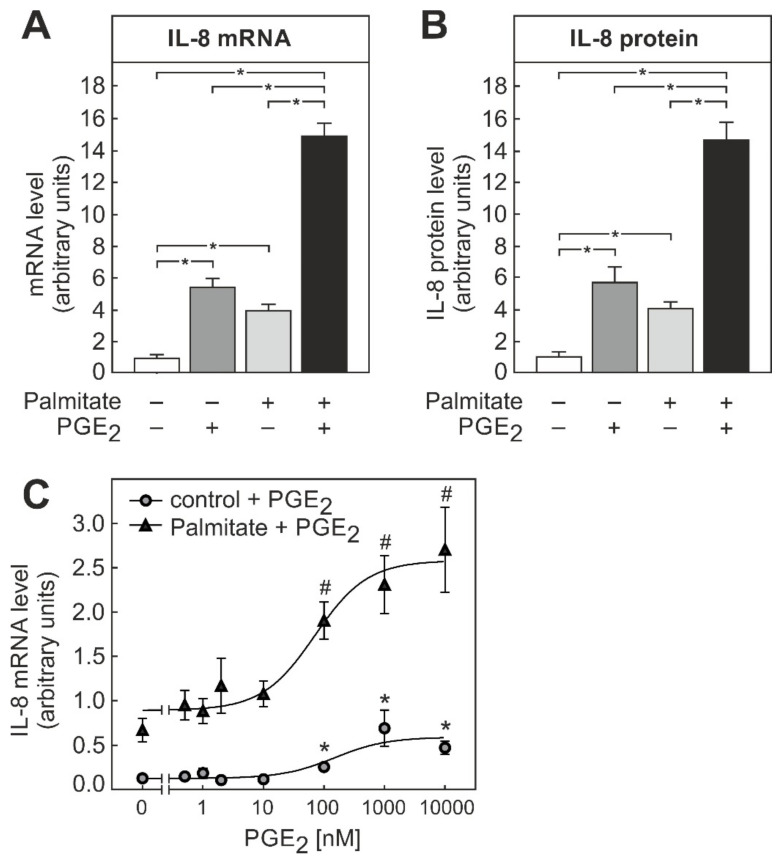
Dose dependence of IL-8 induction by PGE_2_ and palmitate in THP-1 macrophages. THP-1 cells were differentiated into macrophages by PMA treatment, as detailed in the methods section. (**A**, **B**) Macrophages were incubated with 10 µM PGE_2_, 100 µM palmitate, or both for 24 h, as indicated. mRNA levels were determined by RT-qPCR, IL-8 protein was quantified by ELISA. Data were normalized to the average induction under all conditions. Values are means ± SEM of at least 11 independent experiments. Statistics: * *p* < 0.05, two-way ANOVA with Tukey’s post hoc test. (**C**) Macrophages were incubated with the indicated concentration of PGE_2_, 100 µM palmitate, or both for 24 h. IL-8 mRNA levels were determined by RT-qPCR. Values are means ± SEM of 7 to 8 independent experiments per assay point. Significantly different from control 0 nM PGE_2_ in the absence (*) or presence (#) of palmitate *p* < 0.05 in multiple Student’s *t*-tests for unpaired samples.

To elucidate, if PGE_2_ might act in an autocrine feed-forward loop, or by the paracrine activation of neighboring macrophages, the impact of PGE_2_ on IL-8 formation alone or in combination with palmitate was tested. Both PGE_2_ and palmitate significantly induced IL-8 on the mRNA and protein level by roughly 6-fold and 4-fold, respectively (Figure 3A,B). Notably, a more than additive almost 16-fold induction of IL-8 mRNA or protein was observed when THP-1 macrophages were exposed to a combination of PGE_2_ and palmitate.

The impact of PGE_2_ on the IL-8 formation in THP-1 macrophages was dose-dependent. PGE_2_ induced IL-8 mRNA with an EC_50_ of about 150 nM and enhanced the palmitate-dependent induction with an EC_50_ of about 70 nM (Figure 3C). Hence, a significant impact of PGE_2_ on the basal and palmitate-dependent IL-8 expression was already observed at physiologically relevant PGE_2_ concentrations.

### 3.4. Involvement of EP4 Receptor on THP-1 Macrophages in PGE_2_-Dependent Modulation of IL-8 mRNA Induction

**Figure 4:**
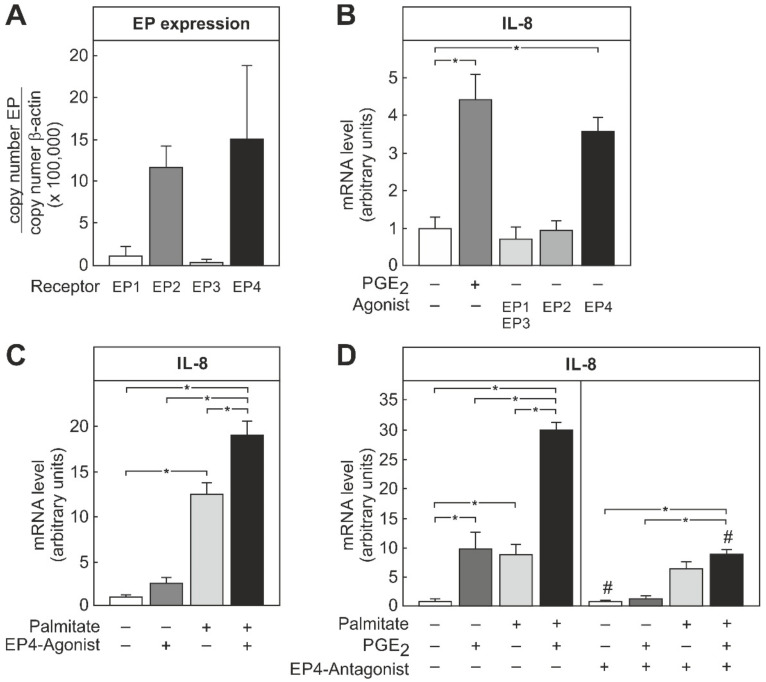
EP receptor expression and EP4-dependent modulation of IL-8 expression in THP-1 cells. THP-1 cells were differentiated into macrophages by PMA treatment, as detailed in the methods section. (**A**) EP receptor expression was determined by RT-qPCR. Copy number was determined by comparison to a plasmid DNA and normalized to the β-actin copy number. (**B**–**D**) Differentiated cells were stimulated with 10 µM PGE_2_, 1 µM of the receptor-specific agonists (EP1/3 agonist: 17-phenyl trinor prostaglandin E_2_; EP2 agonist: 19-(R)-hydroxyprostaglandin E_2_; EP4 agonist: CAY10598), and 100 µM palmitate in the presence or absence of 10 µM of the EP4 receptor-specific antagonist ONO AE3-208, as indicated. IL-8 mRNA was quantified by RT-qPCR. Data were normalized to the average induction under all conditions. Values are means ± SEM of 5 (**A**, **D**) and 3 to 5 (**B**, **C**) independent experiments. Statistics: * *p* < 0.05, # vs. same condition without EP4-Antagonist, one-way ANOVA with Tukey’s post hoc test.

PGE_2_ mediates its action on target cells by four different classes of G protein-coupled receptors—namely, EP1, EP2, EP3, and EP4. To elucidate which of these receptors is responsible for the PGE_2_-dependent modulation of IL-8 expression in THP-1 macrophages, the expression of the different receptors on these cells was first analyzed. Similar to other macrophage populations, the predominant receptor types in these cells were the EP2 and EP4 receptors (Figure 4A). To characterize which of these two receptors is functionally relevant, THP-1 macrophages were stimulated with EP2 and EP4 selective agonists. Only the EP4 agonist was capable of inducing IL-8 mRNA expression to a similar extent as PGE_2_ (Figure 4B). Hence, the EP4 receptor, rather than the EP2 receptor, was involved in the modulation of the IL-8 expression by PGE_2_. Similar to PGE_2_, the EP4 agonist also enhanced the palmitate-induced IL-8 expression in THP-1 macrophages (Figure 4C). In further support of this assumption, the PGE_2_-dependent induction of IL-8 mRNA, as well as the enhancement of the palmitate-dependent IL-8 induction, were completely abolished by an EP4 receptor-specific antagonist (Figure 4D).

## 4. Discussion

The current study showed that the palmitate-induced IL-8 formation in macrophages was increased by the simultaneous presence of insulin (Figure 1). In macrophages, palmitate, especially in combination with insulin, induced the synthesis of PGE_2_ (Figure 2), which also enhanced the palmitate-dependent IL-8 formation (Figure 3), most likely via the EP4 receptor (Figure 4). 

### 4.1. Interaction of Different Factors to Enhance Inflammatory Response

Cells in the chronically inflamed adipose tissue of overweight or obese patients are exposed to a mixture of hormones and metabolites that are elevated beyond the physiological level. Recently, it was shown that high physiological concentrations of insulin elicited an inflammatory response in macrophages [12], and that the combination of LPS and insulin enhanced the inflammatory response over the response obtained by each stimulus alone [11]. While the LPS levels in obese patients or animals under a high-fat diet are probably elevated due to an impairment of the gastrointestinal barrier, insulin resistance in adipose tissue causes an endogenous increase in circulating fatty acids, including palmitate. Initially, adipose tissue macrophages can store fatty acids released from adipocytes as triglycerides without being activated [18]. When this ectopic storage passes a critical threshold, the elevated palmitate concentration may trigger an inflammatory response in macrophages. To compensate for insulin resistance, β-cells increase insulin secretion. The elevated plasma insulin concentration apparently can further increase the palmitate-induced formation of pro-inflammatory cytokines such as IL-8 (Figure 1). IL-8 impairs insulin action on adipocytes and attenuates insulin-dependent Akt-activation [14]; thus, this insulin-dependent augmentation of palmitate-induced IL-8 formation might worsen insulin resistance in a feed-forward loop. The poor utilization of fatty acids in obese patients might further aggravate the problem, since palmitate-induced IL-8 expression enhanced the inhibition of β-oxidation [19], whereas the stimulation of β-oxidation decreased palmitate-dependent IL-8 expression [20]. Finally, IL-8 induction by endogenously released palmitate might also be enhanced by gut-derived LPS [21] or a locally produced tumor necrosis factor α (TNFα) [22]. Thus, the simultaneous presence of elevated concentrations of palmitate, insulin, cytokines, and LPS might act in concert to trigger IL-8 production and foster insulin resistance in a vicious feed-forward cycle. It appears that the simultaneous elevation of insulin and other pro-inflammatory stimuli is essential because insulin delayed and attenuated LPS-activated intracellular signal cascades and IL-8 formation in macrophages when macrophages were exposed to insulin prior to short-term exposure to LPS [23]. In this context, the physiological variations in insulin concentration in healthy subjects with peaks after meals and nadirs in the post-absorptive phase might favor an anti-inflammatory action of insulin, while the continuous hyperinsulinemia in insulin-resistant pre-diabetic patients might favor the pro-inflammatory response.

### 4.2. Possible Role of PGE_2_ in an Autocrine or Paracrine Feed-Forward Loop

Macrophages that were stimulated with a combination of palmitate and insulin released PGE_2_. (Figure 2) PGE_2_, in turn, triggered IL-8 formation and enhanced the palmitate-dependent IL-8 formation (Figure 3A, B). Thus, PGE_2_, similar to the other factors described above, could contribute to a feed-forward augmentation of IL-8 production. Although the existence of such a feed-forward loop can reasonably be assumed to exist in tissues, it was not possible to demonstrate such an autocrine stimulation in the cell culture system used because the absolute concentration of PGE_2_ did not rise to sufficiently high levels in the cell culture supernatants due to the unfavorable cell to supernatant ratio. The highest concentration observed in cell culture supernatants was about 2 nM, an order of magnitude below the EC_50_ (Figure 3C). However, a more than 10-fold higher concentration of PGE_2_ can be expected to occur in the extracellular space in tissues [24,25,26]. The THP-1 macrophages used in this study are but a model for macrophages in vivo. This is a limitation, because the impact of PGE_2_ on macrophage cytokine production may differ between macrophages of different sources and depending on the stimulus. While PGE_2_ did not affect the LPS-induced IL-8 formation in peripheral blood mononuclear cell (PBMC)-derived human macrophages [27], it enhanced the TNFα-dependent formation in PBMCs [28]. Timing might also be a relevant factor. Whereas the simultaneous presence of PGE_2_ with palmitate (Figure 3) or TNFα [28] enhanced IL-8 formation, the incubation of human alveolar macrophages with PGE_2_ prior to subsequent stimulation with LPS inhibited the LPS-induced IL-8 formation in one study [29], whereas it was without effect in another [30]. Finally, PGE_2_ may also inhibit TNFα formation in macrophages [31,32] and Kupffer cells [33], and thereby attenuate the inflammatory response. Currently, it is not clear which of these apparently opposing signaling pathways of PGE_2_ is more relevant in vivo. A recent feeding study in genetically modified mice with impaired PGE_2_ formation suggests that, at least for the development of non-alcoholic steatohepatitis (NASH), the PGE_2_-dependent inhibition of the formation of the pro-inflammatory master cytokine TNFα might be the physiologically most relevant action [25].

### 4.3. EP-Receptor Specificity

The induction of IL-8 formation and the increase in palmitate-dependent IL-8 formation by PGE_2_ were mediated predominantly by the G_s_-linked EP4 receptor (Figure 4). This is in accordance with previous studies which showed that, in human peritoneal macrophages, EP4 antagonists inhibited the PGE_2_-dependent secretion of pro-inflammatory cytokines and chemokines [32,34]. Similarly, in vivo, the knockdown of the EP4 receptor reduced circulating levels of IL-1β and IL-6 in a mouse model of rheumatoid arthritis [35]. In monocytes, the activation of the EP4 receptor enhanced the TNFα-elicited IL-8 formation by activating PKA/CREB/C/EBPβ and NFκB-dependent signal chains [28]. Thus, PGE_2_ via its EP4 receptor may foster the secretion of pro-inflammatory cytokines and chemokines, including IL-8. The EP4 receptor is rapidly desensitized by the phosphorylation of serine residues in its C terminal domain and association with β-arrestin [36]. By contrast, the EP2 receptor confers a sustained activation of G_s_-coupled signal chains in target cells. Most macrophage populations also express the EP2 receptor. While, in the current study, no evidence for a role of the EP2 receptor in the PGE_2_-dependent induction of IL-8 formation was found (Figure 4A,B), others have reported that the EP2 appears to be relevant in the late phase of the PGE_2_-dependent inhibition of TNFα formation [37]. and possibly in the induction of IL-33 formation and the enhancement of LPS-induced IL-1β formation [38,39].

## 5. Conclusions

The current in vitro results suggest that the simultaneous in vivo exposure of macrophages to palmitate and elevated concentrations of insulin, which result from the attempt to compensate for insulin resistance, or prostaglandin E_2_, which is formed in macrophages in response to palmitate and insulin, might enhance palmitate-dependent IL-8 formation and, thereby, aggravate insulin resistance and chronic adipose tissue inflammation in overweight or obese patients.

## Data Availability

The datasets generated and analyzed during the current study are available from the corresponding author on reasonable request.

## Supplementary Materials

The following are available online at https://www.mdpi.com/article/10.3390/biomedicines9050449/s1: Supplementary Table S1: Sequences of the oligonucleotides used for RT-qPCR. Supplementary Table S2: Induction of gene and protein expression of PGE_2_-synthesizing enzymes by individual or combined stimulation with 100 nM insulin and 100 µM palmitate for 24 h in THP macrophages.
