# Supplementary material for: Enhanced Palmitate-Induced Interleukin-8 Formation in Human Macrophages by Insulin or Prostaglandin E2
Source: Biomedicines. 2021 Apr 21;9(5):449. doi: 10.3390/biomedicines9050449 (PMC8143371; doi:10.3390/biomedicines9050449)
Supplement: Supplementary file 1 [file biomedicines-09-00449-s001.zip › biomedicines-1173844-supplementary.pdf]

Enhanced palmitate-induced Interleukin-8 formation in human macrophages by insulin or Prostaglandin E_2

Janin Henkel, Julia Klauder, Meike Statz, Anne-Sophie Wohlenberg, Sonja Kuipers, Madita Vahrenbrink, and Gerhard Paul Püschel

SUPPLEMENTARY MATERIAL

Supplementary Tables

Supplementary Table S1: Sequences of the oligonucleotides (Biolegio, Nijmegen, The Netherlands) used for RT-qPCR. The oligonucleotide sequences were derived on the basis of the given accession numbers. They were checked for specificity by BLAST search.

Gene	Species	Forward (5'-3')	Reverse (5'-3')	Acc. No.
β-Actin	human	CCCCAAGGCCAACCGCGAGAAGATG	AGGTCCCGGCCAGCCAGGTCCAG	NM_001101.3
COX-2 (*PTGS2*)	human	TGTGCCTGATGATTGCCCGACTCC	TGTTGTGTTCCCGCAGCCAGATTG	NM_000963.2
EP1	human	TCGCTTCGGCCTCCACCTTCTTTG	CGTTGGGCCTCTGGTTGTGCTTAG	NM_000955
EP2	human	CGAGACGCGACAGTGGCTTCC	CGAGACGCGGCGCTGGTAGA	NM_000956
EP3	human	CGGGGCTACGGAGGGGATGC	ATGGCGCTGGCGATGAACAACGAG	NM_198712
EP4	human	TCGCGCAAGGAGCAGAAGGAGACG	GGACGGTGGCGAGAATGAGGAAGG	NM_000958.2
IL-8	human	CAGTTTTGCCAAGGAGTGCTAA	AACTTCTCCACAACCCTCTHC	NM_000584.2
mPGES-1 (*PTGES*)	human	GAAGAAGGCCTTTGCCAACCC	GTGCATCCAGGCGACAAAAG	NM_004878.4

Supplementary Table S2: Induction of gene and protein expression of PGE_2-synthesizing enzymes by individual or combined stimulation with 100 nM insulin and 100 µM palmitate for 24 h in THP-1 macrophages. mRNA of microsomal PGE synthase-1 (mPGES1) and cyclooxygenase-2 (COX-2) was quantified by RT-qPCR with β-actin as reference gene. COX-2 protein level was determined in cell lysates and quantified by densitometric analysis of Western blots with fast green staining as a loading control as previously described [11]. Values are means ± SEM of 3 to 6 independent experiments.

* Determination of mPGES1 protein expression was not possible because all commercially available antibodies either failed to reliably detect the protein in Western blots with sufficient sensitivity or were compromised by excessive non-specific cross-reactions.

Statistics: Two-way-ANOVA with Tukey's post hoc test for multiple comparisons; #: $p < 0.05$ versus unstimulated control (K); §: $p < 0.05$ insulin; $: $p < 0.05$ versus Palmitate.

	**K**	**Insulin**	**Palmitate**	**Insulin + Palmitate**
mPGES1 mRNA expression *	1.00 ± 0.24	1.02 ± 0.20	0.89 ± 0.07	1.73 ± 0.16 [#]
COX-2 mRNA expression	1.00 ± 0.18	1.64 ± 0.45	1.07 ± 0.21	2.60 ± 0.35 [# $]
COX-2 protein expression	1.00 ± 0.25	1.67 ± 0.40	2.65 ± 0.81	4.74 ± 0.94 [# §]
